# Austrian Syndrome Associated with Pandemic (H1N1) 2009 in Child

**DOI:** 10.3201/eid1609.091779

**Published:** 2010-09

**Authors:** Waseem Alhushki, Chokechai Rongkavilit

**Affiliations:** Author affiliation: Wayne State University, Detroit, Michigan, USA

**Keywords:** Viruses, influenza, meningitis, endocarditis, pediatrics, Austrian syndrome, letter, *Suggested citation for this article*: Alhushki W, Rongkavilit C. Austrian syndrome associated with pandemic (H1N1) 2009 in child [letter]. Emerg Infect Dis [serial on the Internet]. 2010 Sep [*date cited*]. http://www.cdc.gov/EID/content/16/9/1493.htm

**To the Editor**: In 1957, an American internist reported the preference of *Streptococcus pneumoniae* for the aortic valve and its frequent association with meningitis and pneumonia (*1*), an association now known as Austrian syndrome. This syndrome mainly occurs in middle-age men who have predisposing factors, such as chronic alcoholism, altered immune state, dural fistula, and ear or sinus infections.

One case of Austrian syndrome has been reported in the pediatric age group, in a 7-year-old girl in whom aortic valve endocarditis developed after pneumococcal meningitis infection (*2*). In pneumococcal endocarditis, the native aortic valve is the most frequent location of vegetation. Valve replacement must be considered to avoid development of cardiogenic shock, whereas medical treatment alone may be adequate in some cases of mitral endocarditis (*3*,*4*).

We report a previously healthy adolescent with Austrian syndrome associated with pandemic (H1N1) 2009 infection. Cardiac involvement resulted in extensive mitral valve destruction requiring surgical valve replacement.

A 13-year-old boy had cough, nasal congestion, and a fever >103°F for 2 weeks before being seen at a hospital. On the day he was admitted to hospital, he became unarousable, and weakness was noted on his left side. His medical history included mild asthma requiring no therapy over the past 3 years. His immunizations were up to date except for seasonal influenza and pandemic (H1N1) 2009 vaccination.

When admitted to hospital, his temperature was 38.7°C, blood pressure 97/47 mm Hg, respiratory rate 77 breaths/min, and heart rate 150 beats/min. Pupils were 3 mm in diameter and reactive to light and accommodation. Weakness and hypertonia of the left upper and lower extremities were noted. He had neck stiffness without Kernig sign or Brudzinski sign. Complete blood count showed a leukocyte count of 26,400 cells/mm^3^ and differential of 92.1% neutrophils, 5.3% lymphocytes, 2.6% monocytes, and 0.1% eosinophils; hemoglobin level of 12.9 g/dL; hematocrit 38.6%; and a thrombocyte count of 102,000 cells/mm^3^. A plain chest radiograph showed a left lower lobe infiltrate. Computed tomography of the head showed a large infarction involving the right frontal lobe at the distribution of the right middle cerebral artery; small infarcts involved the left frontal lobe and the right parietal lobe.

Lumbar puncture showed a leukocyte count of 100 cells/mm^3^, 71% neutrophils, 8% bands, 15% lymphocytes, 5% monocytes, and 1% eosinophils; protein 195 mg/dL, and glucose 6 mg/dL, with a blood glucose level of 140 mg/dL. Gram stain of the cerebrospinal fluid showed gram-positive cocci in pairs. The patient was treated with intravenous ceftriaxone, vancomycin, and dexamethasone. He subsequently became unconscious and hypotensive and required intubation with mechanical ventilation and intravenous dopamine. His nasal wash sample was positive for pandemic (H1N1) 2009 RNA by real-time reverse transcription–PCR; oseltamivir (75 mg) through a nasogastric tube every 12 h for 5 days was administered. Because of heart murmur, a 2-dimensional echocardiography was conducted; it showed a large mitral valve vegetation 1.6 cm × 2.1 cm attached to the posterior mitral leaflet and mild to moderate mitral insufficiency. Because of this finding, he was transferred to Children’s Hospital of Michigan for surgical intervention.

Physical examination at Children’s Hospital showed a well-nourished adolescent who was intubated and sedated. A grade 3 systolic ejection murmur at the left lower sternal border was noted. Neurologic examination showed sluggish pupils, decreased tone of the left extremities, and bilateral Babinski sign. No meningeal signs were observed. Magnetic resonance imaging of the head showed multiple areas of infarction, with the largest being in the right middle cerebral artery distribution and a smaller one in the left frontal region (Figure). A few small scattered areas of infarction bilaterally were suggestive of cerebral embolism.

**Figure:**
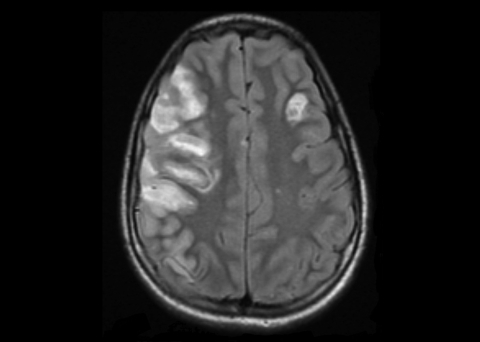
Magnetic resonance image of the head of the patient, a 13-year-old boy, showing multiple areas of infarction bilaterally with the largest in the right middle cerebral artery distribution and a smaller one in the left frontal region consistent with embolic infarcts.

Two days after transfer, the patient underwent mitral valve replacement with a St. Jude prosthetic valve (St. Jude Medical, St. Paul, MN, USA). A 2.5-cm vegetation was found on the atrial surface of the inferior aspect of the posterior mitral leaflet involving the inferior commissure. The posterior leaflet associated with the vegetation was destroyed. The vegetation culture, pericardial fluid culture, tissue culture from the resected mitral valve, and 3 blood cultures yielded no bacterial growth.

The patient required ventilator support for 7 days. Follow-up computed tomography on day 8 showed a stable appearance of cerebral infarcts. Coumadin was started to prevent thrombus development at the prosthetic valve. He went home after completing a 4-week treatment course of ceftriaxone. At that time, there was still noticeable left-sided weakness of the extremities. He could only communicate by using eye movements with no verbal response.

During the 20th century influenza pandemics, secondary bacterial pneumonia was a notable cause of death. The current pandemic (H1N1) 2009 outbreak is evolving rapidly, and it is unknown if pandemic (H1N1) 2009 may lead to an increase in rare complications of pneumococcal infection, such as endocarditis. Thus, Austrian syndrome should be considered in any patient with pandemic (H1N1) 2009 complicated by pneumococcal infection and a new heart murmur.
